# Safety, effectiveness and costs of percutaneous mitral valve repair: A real-world prospective study

**DOI:** 10.1371/journal.pone.0251463

**Published:** 2021-05-12

**Authors:** Iain Willits, Kim Keltie, Mark de Belder, Robert Henderson, Nicholas Linker, Hannah Patrick, Helen Powell, Lee Berry, Julie Speller, Samuel G. Urwin, Helen Cole, Andrew J. Sims

**Affiliations:** 1 The Newcastle upon Tyne Hospitals NHS Foundation Trust, Newcastle, United Kingdom; 2 Translational and Clinical Research Institute, Newcastle University, Newcastle, United Kingdom; 3 National Institute for Cardiovascular Outcomes Research (NICOR), Barts Health NHS Trust, London, United Kingdom; 4 Nottingham University Hospitals NHS Trust, Nottingham, United Kingdom; 5 South Tees Hospitals NHS Foundation Trust, Middlesbrough, United Kingdom; 6 National Institute for Health and Care Excellence, London, United Kingdom; 7 National Institute for Health and Care Excellence, Manchester, United Kingdom; 8 National Casemix Office, NHS Digital, Leeds, United Kingdom; Maastricht University Medical Center, NETHERLANDS

## Abstract

**Aims:**

Percutaneous mitral valve leaflet repair is a treatment option for some people with severe mitral valve regurgitation for whom conventional mitral valve surgery is clinically inappropriate. This study aimed to determine the safety, efficacy, and costs of percutaneous mitral valve leaflet repair, using the MitraClip device in a UK setting.

**Methods and results:**

This was a prospective, single-armed registry with a follow-up of 2 years that reported a range of procedural, clinical and patient-orientated outcomes. Registry data were linked to routine data sources to allow for more comprehensive follow up concerning mortality and healthcare resource use. The registry received data for 199 mainly elective patients with mixed mitral regurgitation aetiology. A MitraClip device was implanted in 187 patients (94%), with a procedural success rate of 86%, with 8% of patients having a serious in-hospital adverse event (including 5% mortality). Percutaneous mitral valve leaflet repair reduced mitral regurgitation from 100% MR grade ≥ 3+ to 7% at discharge. There were corresponding improvements in New York Heart Association functional class, reducing from 92% (class ≥ 3) at baseline to 18% at 6 weeks. There were significant improvements in generic and disease specific quality of life indicators up to 2 years. The all-cause mortality rate was estimated to be 12.7% (95% CI 7.5 to 17.7%) at 1 year. Percutaneous mitral valve leaflet repair was associated with reduced hospital readmissions and potential cost-savings in post-procedural care.

**Conclusion:**

This study shows that percutaneous mitral valve leaflet repair using MitraClip is a relatively safe and effective treatment in patients unable to tolerate surgery and has the potential to reduce ongoing healthcare costs in the UK.

## Introduction

Mitral valve regurgitation (MR) is a significant cause of morbidity and mortality in older people. In the UK, it is estimated that 9.4% of the adult population have MR, with 0.5% [1] severe. Usually, MR is initially asymptomatic, but manifests with palpitation, dyspnoea, exercise-induced fatigue and ankle swelling as it progresses [2] and is associated with a diminished quality of life and reduced life expectancy [3].

MR is normally classified as degenerative (DMR, primary or structural, MR) when the valve itself deteriorates, or functional (FMR, secondary MR), when the cause is secondary to structural changes of the ventricles, as can occur following ischaemic damage, cardiomyopathy, or related diseases. Rarely, a mixed aetiology can occur. Although open surgery is often indicated for severe DMR [4], and less commonly for FMR [5], the risks of surgical intervention can sometimes outweigh the benefits in patients with advanced MR.

In the 2000s, research on percutaneous edge-to-edge mitral valve repair (PMVR) techniques led to the development of the MitraClip^™^ device (Abbott Laboratories, IL, US) [6, 7] to reduce the leak through the valve. For patients ineligible for surgery, PMVR may represent the only feasible alternative to conservative medical treatment, including palliation.

In 2013, PMVR was included within the NHS England Commissioning through Evaluation (CtE) Programme [8]. This allowed a limited number of patients to access procedures not routinely commissioned, whilst prospective safety and efficacy data were collected. The aims of this study are to assess the efficacy, safety and costs in the UK of PMVR in patients with severe MR considered to be at prohibitive risk of surgery.

## Materials and methods

### Design and ethics

This was a prospective observational study using a registry [9] to capture characteristics of patients, procedures and outcomes, with linkage to two administrative datasets (Hospital Episode Statistics [HES] and Office of National Statistics [ONS]). Follow up was scheduled at 6 weeks, 6 months, 1 year, and 2 years for a range of clinical outcomes and patient-reported outcome measures (PROMs). Mortality data were extracted from the ONS after linkage using a third party (NHS Digital). Results were reported using STROBE criteria [10].

Patients gave written informed consent to receive PMVR through the NHS England CtE programme. Approvals for data collection, data linkage and analyses were granted by the NHS Health Research Authority Confidentiality Advisory Group (Ref: 17/CAG/0153, CAG 10-07(b)/2014) and NHS Digital (Ref: DARS-NIC-151212-B5Z3R). Participating hospitals provided data to the National Institute for Cardiovascular Outcome Research (NICOR) and data were then analysed by the National Institute for Health and Care Excellence (NICE) External Assessment Centre at the Newcastle upon Tyne Hospitals NHS Foundation Trust.

### Patient and public involvement

Patients and/or the public were not involved in the design, conduct, reporting or dissemination plans of this research.

### Patient selection, follow-up and outcomes

Four centres in England contributed data to the registry. Patients with MR and heart failure considered eligible for surgical mitral valve replacement or repair, or for PMVR, were referred from secondary care to cardiac surgeons or cardiologists in specialist cardiac centres. Patients were selected for suitability for PMVR at multidisciplinary team (MDT) meetings according to defined eligibility. Patients were required to have symptomatic severe grade 3+ or 4+ MR; be at excessive risk for conventional mitral valve surgery; have suitable cardiac anatomy; and be able to give informed consent. Funding was made available from NHS England for the inclusion of 180 patients in the registry.

Procedural and in-hospital data were collected to determine safety, efficacy, and use of healthcare resources. Efficacy outcomes included procedural success, change in MR grade, mortality and PROMs. The New York Heart Association (NYHA) functional class was measured to assess severity of heart failure (dyspnoea) symptoms and the 6 minute walk test to determine exercise capability. Health-related quality of life (HRQoL) data were collected using EQ-5D-5L (EuroQol), and the Kansas City Cardiomyopathy Questionnaire (KCCQ) [11]. Safety outcomes included major and minor complications in hospital and after discharge. The economic measures were readmission rates, total length of stay and healthcare resource usage, in UK pounds sterling. Full definitions of each outcome are given in S1 Table.

### Data linkage

Identifiable data from patients were linked with HES and ONS by NHS Digital, utilising an eight step matching algorithm based on NHS number, date of birth, gender, postcode, and study number [12]. Data from HES included all inpatient finished consultant episodes from matched patients with hospital discharge dates between 1st April 2008 and 1st March 2018. Data from ONS included all deaths from matched patients reported until 1st March 2018. Outcomes of interest were mortality and requirement for additional mitral valve intervention. Linked records with conflicting demographic and administrative details were used to indicate potential matching errors and excluded from subsequent long-term analysis.

### Economic analysis

“Before” and “after” healthcare resource usage was extracted from linked data, following the methodology of a prior US study [13]. HES was used to identify hospital admissions in the years prior to and following the procedure, and costs were derived from the Healthcare Resource Group (HRG) code associated with each admission. HRG codes are groupings of patient events that have been judged to consume a similar level of resource, used in the NHS for reimbursement (S1 Protocol).

### Statistics

Statistical summaries and tests were performed using the programming language R [14]. Univariate analyses, with Bonferroni correction for multiple tests, were applied to each outcome measure to test for significant association with each explanatory variable. Multivariate tests were applied to each outcome measure, with associations reported as odds ratios (OR) with 95% confidence intervals (CI).

Severity of MR recorded in the registry at each follow-up point was compared with pre-operative severity using Fisher’s exact test (paired analysis). Paired quality of life scores and utilities recorded in the registry were compared at each follow up point against pre-operative scores using Fisher’s tests or t-tests where appropriate. Kaplan-Meier analysis was applied to mortality outcomes from linked data. For the economic analysis, rate ratios and their 95% CIs were estimated with generalised log-Poisson multilevel models [13].

## Results

**Fig 1:**
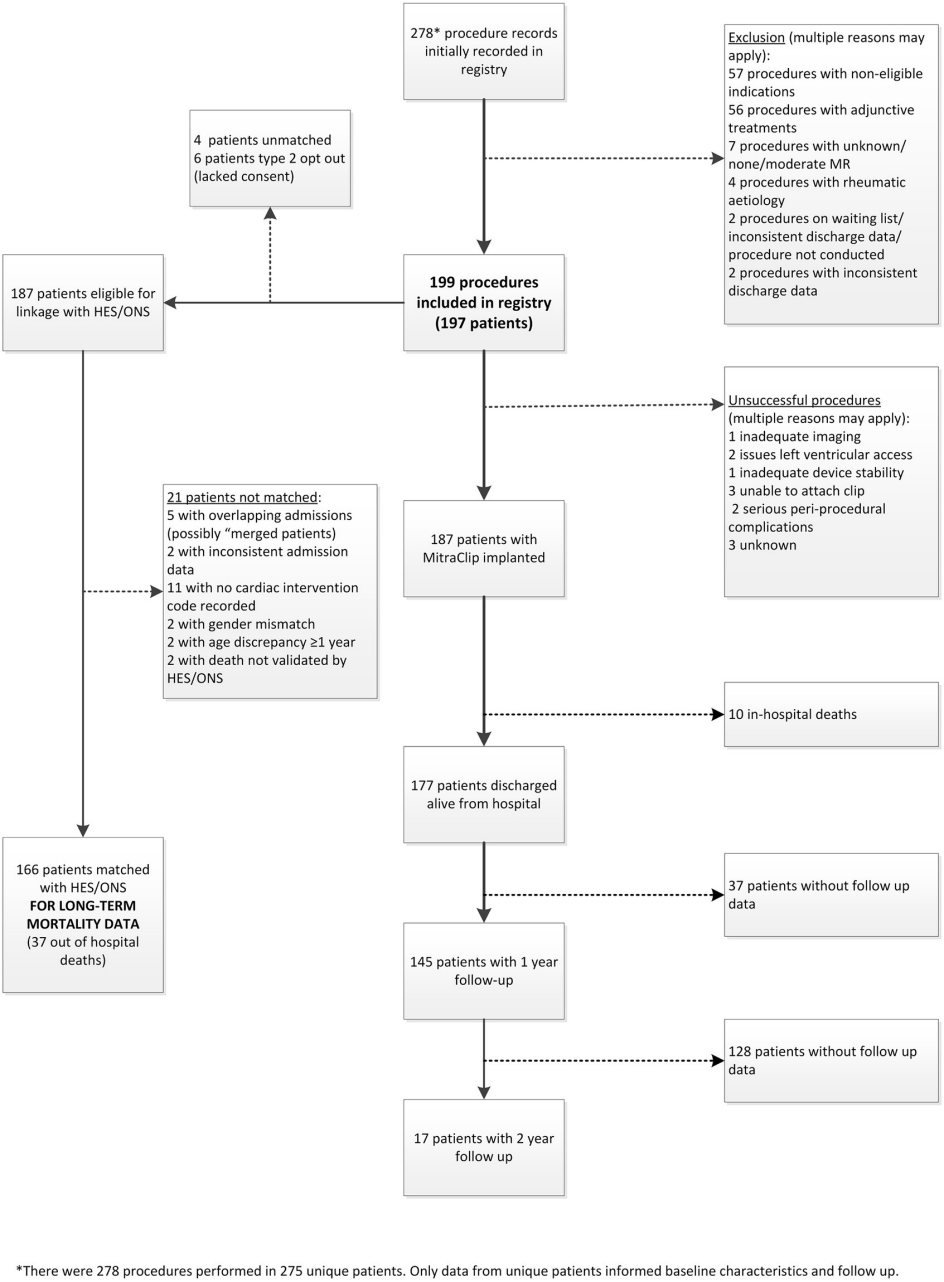
Patient flow in the CtE registry.

A total of 278 PMVR procedure records, from 275 patients, were recorded in the registry between October 2014 and April 2018, of which 199 PMVR procedures from 197 patients (2 patients had a repeat procedure) were eligible for analysis of in-hospital outcomes (Fig 1). Procedures were excluded (n = 57) if the reason for treatment did not include either surgical turn-down, high-risk for surgery (bail-out surgery would be offered) or high-risk for surgery (bail-out surgery would not be offered); thus procedures were excluded if the reason was documented as patient preference, clinician preference, or other. A further 56 procedures with adjunctive treatments were excluded if the plan for treatment did not include stand-alone MitraClip procedure; or was with PCI. Thus procedures were excluded if the plan for treatment was documented as with “procedure on atrial septum”, “other valve procedure”, “surgical procedure”, “medical treatment” or “other”.

A total of 187 patients were linked by NHS Digital to HES and/or ONS, of which 21 (11.2%) had conflicting demographic fields and were excluded. A total of 166 PMVR procedures were included in follow-up outcome analysis.

Patient characteristics are reported in Table 1. Nearly all patients had moderate or severe MR (99.5% grade ≥ 3); most (60%) had FMR aetiology. This was reflected by the dyspnoea status of the patients, with 92.4% having an NYHA class of ≥ 3. Nearly all were at high risk for surgery (median logistic EuroSCORE II of 4.8, interquartile range [IQR] 3.0 to 7.6, range 0.7 to 42.5).

**Table 1:** Procedural characteristics in the registry.

Characteristic	All procedures (n = 199)^a^
Gender	
• Female, n (%)	62 (31.2%)
Age (years)	
• Median	78.5
• (Q1, Q3) [range]	(69,84.8) [37–94]
MR aetiology	
• Functional/ischaemic	117 (60.0%)
• Degenerative	78 (40.0%)
Severity of mitral regurgitation	
• Grade 2 (mild to moderate)	1 (0.5%)
• Grade 3 (moderate to severe)	20 (10.1%)
• Grade 4 (severe)	178 (89.4%)
LVEF	
• Good (≥ 55%)	66 (39.5%)
• Mild impairment (45–54%)	31 (18.6%)
• Moderate impairment (30–44%)	39 (23.4%)
• Severe impairment (<30%)	31 (18.6%)
Estimate of TR severity	
• No TR	14 (8.6%)
• Mild	75 (46.0%)
• Moderate	50 (30.7%)
• Severe	24 (14.7%)
BNP (pre-op median), pg/ml	
• median	376
• (Q1,Q3) [range]	(162,846) [13–2000]
Logistic EuroSCORE	
• median	15.6
• (Q1,Q2) [range]	(9.6,27.1) [1.9–74.7]
Logistic EuroSCORE II	
• median	4.8
• (Q1,Q2) [range]	(3.0,7.6) [0.7–42.5]
Critical pre-op status	5 (2.6%)
Risk factors	
• Diabetes	36 (18.1%)
• Hypertension	104 (53.3%)
• Previous neurological disease	26 (13.1%)
• Peripheral vascular disease	28 (14.4%)
• Previous myocardial infarction	79 (40.3%)
• Angina pectoris	49 (25.0%)
• Non-sinus rhythm	117 (59.4%)
• Previous PCI	45 (23.1%)
• Previous cardiac surgery	78 (39.6%)
• Severe liver disease	1 (0.5%)
• History of pulmonary disease	51 (26.0%)
• Previous electric device therapy	51 (25.8%)
NYHA dyspnoea status	
• No limitation of PA	3 (1.5%)
• Slight limitation of ordinary PA	12 (6.1%)
• Marked limitation of ordinary PA	124 (62.6%)
• Symptoms at rest or minimal PA	59 (29.8%)
6 minute walk test, m	
• Median	190
• (Q1,Q3) [range]	(108,294) [0,450]

Abbreviations: BNP, B-type natriuretic peptide; LVEF, left ventricular ejection fraction; MR, mitral valve regurgitation; NYHA, New York Heart Association; PA, physical activity; Q, quartile; TR, tricuspid valve regurgitation.

^a^Statistical analysis reported no significant difference between eligible cohort, implanted cohort, and cohort linked with HES/ONS datasets for any characteristic.

Procedural information is reported in Table 2. In the majority of cases (84.4%) PVMR was elective (routine admission from waiting list), with 13.6% urgent (on waiting list but PMVR moved forward), and 2.0% an emergency (unscheduled). The technical success rate (device successfully implanted) was 94.0% (95% CI 89.7 to 96.8%), and procedural success rate (technical success in absence of major adverse events) was 85.9% (95% CI 80.3 to 90.4%). Most procedures required two or more clips to be implanted (69.0%) and the median procedural duration was 180 minutes (IQR 137 to 221). There were no peri-procedural deaths, although 10 patients (5.1%) died in hospital before discharge. Major adverse events occurred in 16 procedures (8.2%, 95% CI 4.7 to 12.9%) in hospital, and 15 were associated with a minor adverse event in hospital (7.6%, 95% CI 4.3 to 12.2%). Additional information is available in S2 Table.

**Table 2:** Procedural details and in-hospital complications.

Characteristic	All procedures (n = 199)
Urgency	
• Elective	168 (84.4%)
• Urgent	27 (13.6%)
• Emergency	4 (2.0%)
Patients with device implanted	187 (94.0%)
No. of clips opened	
• 1	54 (28.0%)
• 2	111 (57.5%)
• 3	24 (12.4%)
• 4	4 (2.1%)
No. of clips successfully deployed	
• 1	58 (31.0%)
• 2	103 (55.1%)
• 3	26 (13.9%)
Procedural success rate ^a^	171 (85.9%)
Fluoroscopy time, mins	
• Median	32
• (Q1:Q3) [range]	(22,43) [8–79]
X-ray dose, mGray.cm^2^	
• Median	3879
• (Q1:Q3) [range]	(3000,6948) [3000–20,000]
Contrast dose, ml	
• Median	0
• (Q1:Q3) [range]	(0,0) [0–105]
Procedural duration, mins	
• Median	180
• (Q1:Q3) [range]	(137,221) [54–300]
Time from procedure to extubation, mins	
• Median	210
• (Q1,Q3) [range]	(160,276) [72–10140]
Required ITU stay	38 (31.4%)
Length of stay, days	
• Median	5
• (Q1:Q3) [range]	(3.25,8) [0,46]
In-hospital deaths	10 (5.0%)
In-hospital AE	
• Major ^b^	16 (8.2%)
• Minor ^c^	15 (7.6%)
• Any	30 (15.2%)

Abbreviations: AE, adverse event; ITU, intensive therapy unit; no., number; Q, quartile.

^a^ Procedural success was the device successfully implanted in the absence of major complications.

^b^ Major complications were: death (10); neurological event (1); additional surgery (3); device embolisation with percutaneous retrieval (1); myocardial infarction (2); endocarditis (0); pericardial effusion/tamponade requiring intervention (0); major vascular injury requiring intervention (0); mitral valve complication (0); oesophageal rupture (1); major bleed (3); stage 2/3 acute kidney injury (4); cardiogenic shock (2).

^c^ Minor complications were: device failure (0); partial detachment (1); pericardial effusion/tamponade, treated conservatively (3); thrombus (0); new moderate/severe mitral stenosis (3); minor bleed (7); stage 1 acute kidney injury (1); minor vascular complication (0).

**Fig 2:**
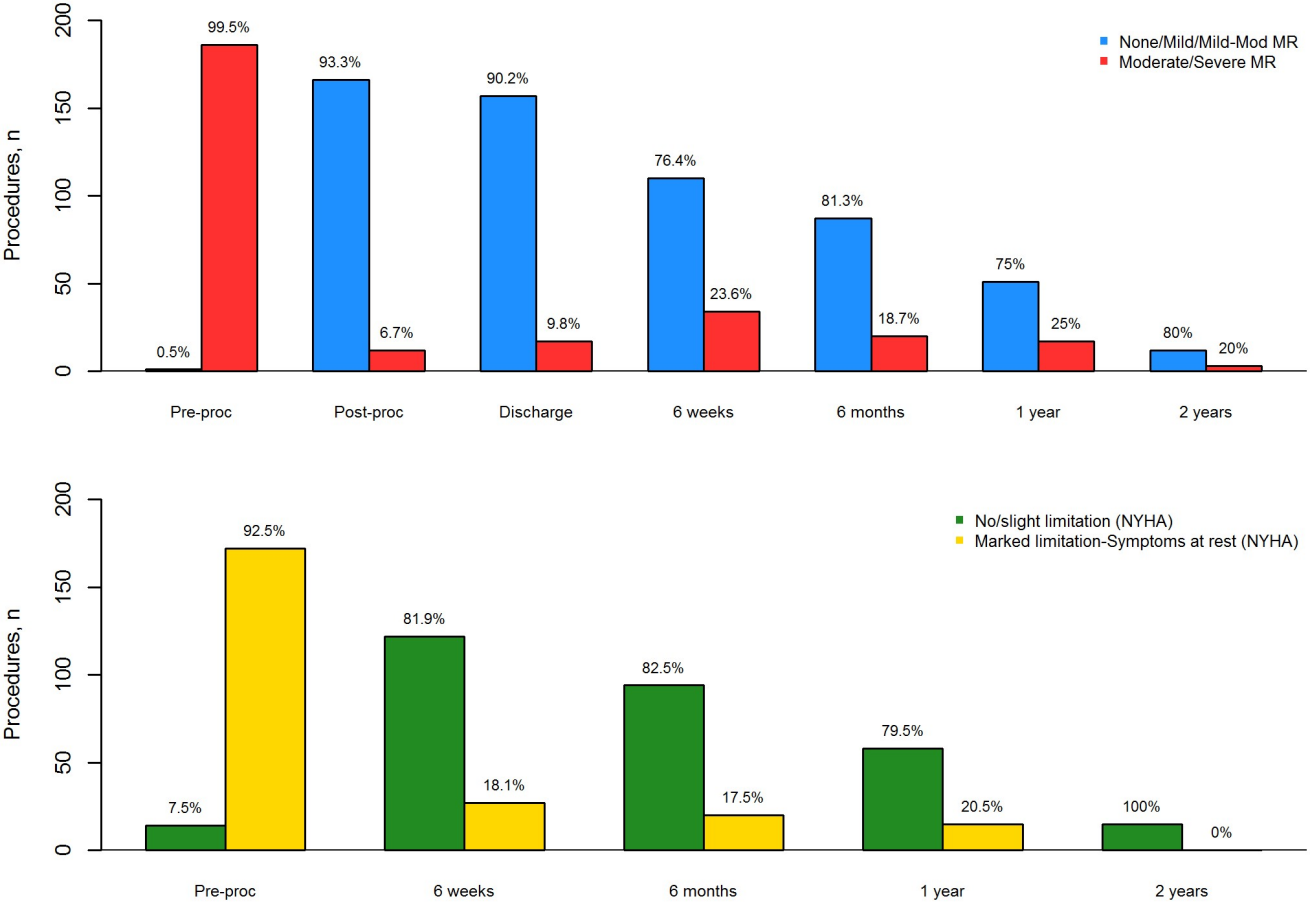
Dichotomised MR grade (a) and NYHA class (b) over 2 years follow up.

There was significant reduction in MR at discharge and later time points compared with baseline (p < 0.001, S3 Table). Following PMVR, the majority of patients (93%) were classified as MR grade ≤ 2 on discharge from hospital. This effect persisted at time points up to 2 years (Fig 2a). The reduction in MR grade was mirrored by improved dyspnoea symptoms (Fig 2b), with only 18% having marked or severe symptoms of dyspnoea 6 weeks after discharge. Improvements in dyspnoea were observed throughout the 2-year follow up of the study (S4 Table).

**Fig 3:**
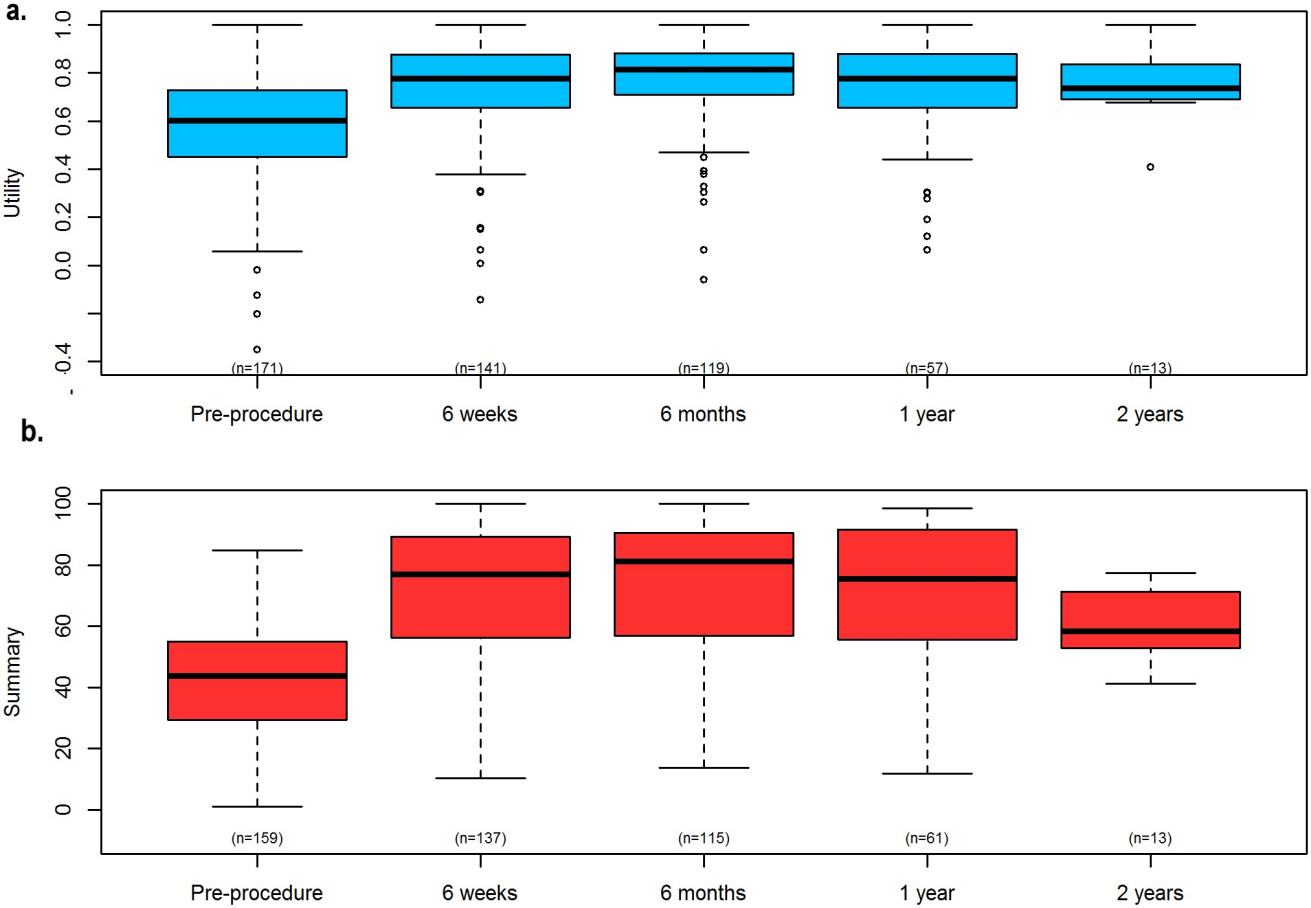
Aggregate EQ-5D utility scores (a) and overall summary KCCQ scores (b) over 2 years follow up.

Median baseline generic health-related quality of life (HRQoL) in 163 procedures was 0.60 (IQR 0.45 to 0.72). The mean baseline utility score was 0.55 (SD 0.23). There was a statistically significant improvement in HRQoL observed at 6 weeks (mean difference 0.18 (SD 0.23), p < 0.001, n = 136), and 113 (83.1%) showed an improvement in utility, with 9 (6.6%) showing no change. This improvement was sustained throughout the study (Fig 3a). Analysis of individual EQ-5D domains (mobility, self-care, usual activities, pain/discomfort and depression/anxiety) showed trends for improvement at all time-points, which were mostly statistically significant up to 1 year. Additionally, there were more patients reporting improved rather than reduced utility compared with baseline at all follow up time points. Improvements in HRQoL utility were supported by changes in the visual analogue score (VAS), measured as 50 mm (IQR 35 to 65 mm, n = 141) at baseline, and 70 mm (50 to 80 mm, n = 105), 70 mm (60 to 80 mm, n = 88) and 75 mm (55 to 85 mm, n = 52) at 6 weeks, 6 months and 12 months respectively (S5 Table). KCCQ scores also showed significant improvements in disease-specific HRQoL; the pre-procedural mean (SD) summary score was 37.7 (19.4) (median 41.7, IQR 25.0 to 50.0) compared with a mean (SD) of 70.9 (28.1) (median 83.3, IQR 58.3 to 91.7) in 57 patients with paired recordings at 1 year (Fig 3b, S6 Table).

**Fig 4:**
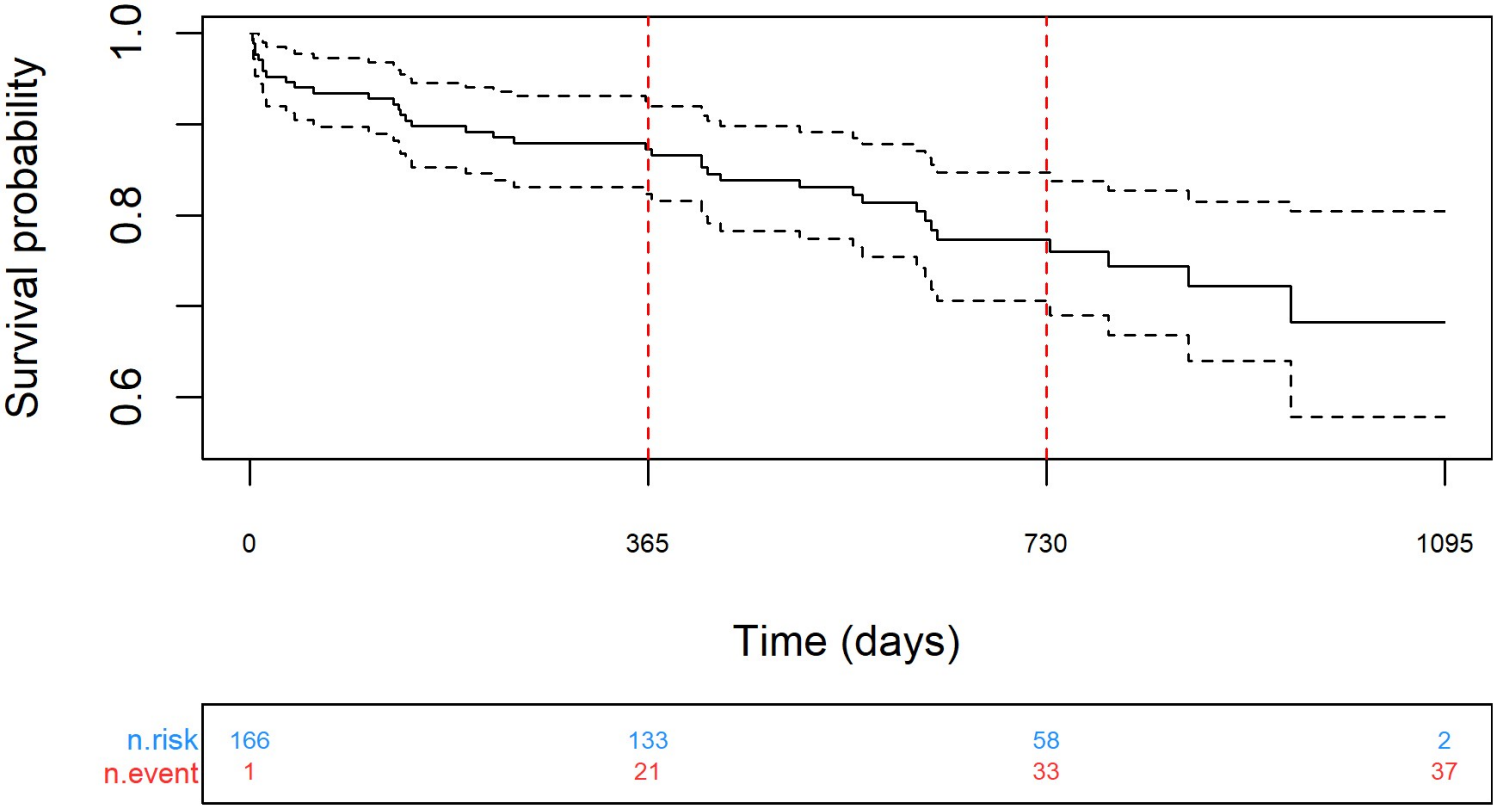
Kaplan-Meier analysis of mortality over 2 years follow up.

The all-cause mortality rate was 12.7% (95% CI 7.5 to 17.7%) at 1 year (n = 133), and 22.7% (95% CI 15.3 to 29.4%) at 2 years (n = 58) (Fig 4); median follow-up was 610 days (IQR 409 to 807). In the year prior to PMVR, there were 470 admissions to hospital. In the year after the procedure, there were 251 admissions (rate ratio 0.57, 95% CI 0.49 to 0.67, p < 0.001). No significant difference in mortality was detected between patients with FMR or DMR, or patients treated electively or urgently; however, the study was not designed to detect differences (S1 Fig). There were also decreases in total days spent in hospital and associated aggregated costs, which overall were reduced in a cohort of 150 patients from approximately 1,267,000 GBP pre-procedure to 890,000 GBP after PMVR (Table 3). This reduction in healthcare resource use was mainly related to a reduction in admissions due to cardiac reasons, and in particular heart failure indications. Conversely, there was an increase in non-cardiac admissions observed, although this did not offset the overall cost savings (S7 Table).

**Table 3:** Hospital resource use before and after PMVR.

Outcome	Pre MVR	Post PMVR	RR (95% CI)	P
*All patients*				
Admissions	470	251	0.57 (0.49 to 0.67)	<0.001
Days in hospital	1635	1340	0.73 (0.54 to 0.98)	N/A
Total costs (GBP)	1,267,321	889,721	0.62 (0.50 to 0.79)	N/A
*Surviving patients*^a^				
Admissions	418	221	0.53 (0.45 to 0.62)	<0.001
Days in hospital	1432	1092	0.67 (0.49 to 0.93)	N/A
Total costs (GBP)	1,130,425	743,260	0.57 (0.45 to 0.73)	N/A

Abbreviations: GBP, British pound sterling; N/A, not applicable; RR, rate ratio.

^a^Sensitivity analysis was performed where patients who had died post-procedure were excluded from analysis. This was done to address the “positive” economic consequences of healthcare resource reduction due to death, bearing in mind the high mortality rate associated with the procedure.

## Discussion

Evidence on the use of PMVR in the UK is limited to a European registry study [15]. Our study was a multicentre, prospective, observational registry, with consecutive patient selection through MDT assessment and treatment, reflecting real-world practice in the UK. We report extensive clinical outcomes, including those most valued by patients (PROMS and HRQoL). We assessed healthcare resource usage, in a UK setting, by linking to HES and ONS The results of this study have been used to inform the future national commissioning policy for PVMR within the NHS.

Our key findings were as follows. Firstly, PMVR using the MitraClip system was successfully implanted in 94% of cases, with a procedural success rate of 86% and in-hospital death rate of 5%. Secondly, PMVR was associated with a statistical and clinically significant improvement in MR, which was sustained to 2 years. Thirdly, improvements in MR were reflected in significant improvements in PROMs including symptoms (NYHA class), generic HRQoL and disease-specific HRQoL. Fourthly, despite these clinical improvements, the medium term mortality rate remained high, reflecting the high level of morbidity in this population. Finally, PMVR was associated with a decrease in use of hospital resources, highlighting the potential for future cost savings.

Our study indicated that the use of PMVR in this cohort has the potential to reduce hospital resource costs overall and specifically for cardiac causes in the following year, and possibly beyond. Comparing the year pre- and post- procedure, there was a greater reduction in the rate of all-cause admissions (rate ratio 0.57; 95% CI 0.49 to 0.67) than reported by Vemulapalli et al. in a comparable US registry study [13] (RR 0.82; 0.73 to 0.92); rate ratios among those surviving to 1 year were similar (0.53; 0.45 to 0.62, versus 0.60; 0.52 to 0.68). There was a significant reduction in the total days admitted (RR 0.73; 0.54 to 0.98), in contrast to the US study which reported an increase in the all-cause rate of days in hospital (RR 1.36; 1.29 to 1.42) for the whole cohort, although the rate ratios were similar in the surviving cohort. The age and MR aetiology in the two studies was similar (S8 Table), but the cohort in this study had worse MR grade (99.5% grade 3,4 versus 85.1%) and worse NYHA class (92.4% class III, IV, versus 83.4%) than the US registry study [13], suggesting greater potential for hospital resource saving than previously reported. However, the study did not consider cost of the procedure itself and did not assess the cost-effectiveness of PMVR. A previous economic analysis has indicated that MitraClip has the potential to be cost-effective at UK willingness-to-pay thresholds [16], but this analysis was based on the extrapolated long-term data from the EVEREST HRS study [17]. The recent randomized trials [18, 19] may have the potential to inform more accurate cost-effectiveness data in patients with FMR.

Nearly all published research into PMVR has been carried out using the MitraClip system. The first comparative study on PMVR was the EVEREST II randomised controlled trial (RCT), which compared PMVR with open surgical techniques in patients with DMR and FMR [20], with further results at 5 years [21]. Whilst the primary outcome of EVEREST II did not show non-inferiority, the initial EVEREST studies were conducted in populations who were relatively healthy with limited comorbidities and, by definition, were candidates for surgery. Large observational studies in non-surgical candidates followed [15, 22–24], but did not report comparative evidence. More recently, two RCTs have been published in patients with FMR and not suitable for surgery. The MITRA-FR study [18] and the COAPT trial [19] both compared PMVR with medical management, but only the latter reported positive outcomes in terms of improved mortality and reduced rehospitalisation rates, emphasising the need for careful patient selection [25]. Thus, the evidence base for PMVR is now substantial, although still relatively lacking in patients with DMR aetiology.

The results of our study are consistent with other studies. Although a MitraClip device was successfully attached in 94% of patients, the procedural success rate was 86%. This was due to an early major adverse event rate of 8%, including 10 in-hospital deaths (5%) after the procedure. This seems consistent with other studies, and reflects the high level of morbidity in the PMVR population. As reported in all other studies of PMVR, the procedure is associated with a large improvement in mitral valve functionality. However, the mortality rate is high in the first 2 years, with our study observing a death rate of 12.7% at 1 year and 22.7% at 2 years. It is unknown whether PMVR reduced mortality, because there was no comparator group. However, our results concur with previously results, and mortality was lower than the 1 year mortality rates observed in the intervention arms of the two recent RCTs [18, 19]. Data from the COAPT study suggests mortality may be reduced at 2 years in patients with FMR who are carefully selected [19]. However, there were some potentially important differences between the study cohorts (S8 Table) which limit their comparability. The patients in our study were slightly older than those in the MITRA-FR study [18], but had fewer comorbidities (fewer with diabetes, fewer previous MI, lower surgical risk), less severe disease (greater LVEF) and had mixed aetiology (40% DMR). In comparison with the COAPT cohort, patients in our study were older, had fewer comorbidities (fewer with diabetes, fewer with previous MI or PCI), more severe disease (worse MR grade) and mixed aetiology.

Our study demonstrated unequivocally that PMVR relieves symptoms of heart failure, most notably dyspnoea. We also reported significant gains in HRQoL overall, and in all domains of the EQ-5D standardised system. The mean (SD) change in utility score at 12 months was 0.15 (0.22) and the mean change in visual analogue scale (VAS) was 30 mm, which are regarded as important patient-orientated improvements [26]. Significant gains in disease-specific HRQoL (KCCQ) were observed and mirror improvements in HRQoL in other studies. The TRAMI registry reported significant improvement in EQ-5D-3L VAS and the domains of self-care and anxiety/depression at 1 year follow up [27]. Studies have also reported both physical and mental improvements HRQoL using the Short Form 36 (SF36) system [28] and the KCCQ [19].

The study had some limitations. The registry was single-armed, and comparisons were with previous studies [29]. The number of patients receiving optimized heart failure medical therapy was not recorded. Hospital resource costs were available for in-patient admissions (including day-cases) which represent the majority of costs, but not for out-patient or primary care consultations. Although a placebo effect cannot be excluded, the inclusion of hard physiological outcomes and reporting of longitudinal PROMs provide objective evidence of the impact of PMVR. Because the results relate to the early experience with this device in the participating hospitals, a core lab was not used to evaluate findings from echocardiography and other procedures. There was loss to follow-up as some patients attended appointments in referring hospitals rather than the specialist centre. The duration of follow up was only 2 years, and most patients were not eligible for assessment at this time point because of the timeframe of the study. Our unit of assessment was procedures, not patients, leading to potential loss of statistical independence. However, only two patients had repeat procedures (after 10 and 17 months) and the effect on results is likely to be negligible in comparison with other grouping factors, such as the treating hospital. Whilst data linkage allowed for more complete mortality follow up, it did not allow for more complete coverage of other important outcomes, such as MR grade and HRQoL. Additionally, this study reported on a case mix of patients with, for instance, different valve aetiology or procedural urgency. These cohorts were analysed in aggregate due to the relatively small sample size, and subgroup analysis was not statistically viable. Thus the reported outcomes may not be representative of individual groups, but as recent trial evidence has shown, patient selection is likely to be crucial in maximising the clinical and cost-effectiveness benefits of this treatment.

## Conclusions

This is the first study of its type performed solely in a UK NHS setting. The results from the study indicate that the PMVR procedure is not without risk, reflecting the high morbidity burden of the treated population. Procedural success rate, adverse events, and mortality rates over time were consistent with other studies, as was the significant improvement in MR grades and NYHA class. In addition, our study showed that PMVR is associated with a significant improvement in generic HRQoL in surviving patients and has the potential to reduce ongoing hospital costs. Overall, the results of this study are concordant with previous studies and demonstrate the feasibility of PMVR in the UK NHS setting.

## Supporting information

S1 Protocol Detailed description of economic analysis. (DOCX)

S1 Table List of outcome measures used in the registry. (DOCX)

S2 Table Adverse events (in hospital and following discharge). (DOCX)

S3 Table MR grade over 2 years follow up. (DOCX)

S4 Table NYHA class over 2 years follow up. (DOCX)

S5 Table S5a. HRQoL data (EQ-5D) over 2 years follow up. S5b. EQ-5D VAS over 2 years follow up.
(DOCX)

S6 Table HRQoL disease-specific data (KKCQ) over 2 years follow up. (DOCX)

S7 Table Hospital resource use before and after PMVR, stratified by reason for readmission. (DOCX)

S8 Table S8a. Comparison of demographics, inclusion and exclusion criteria with the MITRA-FR study [18]. Shaded rows indicate potentially important differences. S8b. Comparison of demographics, inclusion and exclusion criteria with the intervention arm of the COAPT study [19]. Shaded rows indicate potentially important differences. S8c. Comparison of demographics, inclusion and exclusion criteria with the EVEREST II/REALISM Continued Access Registry [13]. Shaded rows indicate potentially important differences.
(DOCX)

S1 Fig Kaplan-Meier analysis over 2 years follow up of: (a) patients receiving elective treatment; (b) patients receiving urgent or emergency treatment. (DOCX)

S2 Fig Kaplan-Meier analysis over 2 years follow up of: (a) patients with functional MR; (b) patients with degenerative MR. (DOCX)
