# Supplementary material for: Safety, effectiveness and costs of percutaneous mitral valve repair: A real-world prospective study
Source: PLoS One. 2021 May 12;16(5):e0251463. doi: 10.1371/journal.pone.0251463 (PMC8115844; doi:10.1371/journal.pone.0251463)
Supplement: S1 Protocol — (DOCX) [file pone.0251463.s001.docx]

# Materials and methods

## Economic analyses

We calculated hospitalisation costs by converting each finished consultant episode of the linked HES APC extract to Healthcare Resource Groups (HRGs) by applying the HRG4+ 2017/18 Reference Costs Grouper [National Casemix Office]. We referred to the National Schedule of Reference Costs 2017/18 at aggregate level using the mean costs from all NHS trusts and NHS Foundation trusts [NHS Improvement]. We applied this process to all years of data to give a consistent cost baseline for comparative purposes (noting that grouping rules and reference cost do vary annually). Therefore this approach provides an approximation the costs for the activity as recorded in the Reference Costs collection, which is useful for comparative analysis (before and after MitraClip implantation) but will not give a robust estimate of absolute total cost saving or expenditure.

To use the Reference Costs Grouper, we categorized each episode in the HES by admission type:

- day case (CLASSPAT=2), regular day or night (CLASSPAT=3 or 4);
- elective inpatient (CLASSPAT=1 and ADMIMETH=11,12,13,81);
- non-elective short stay (ADMIMETH=21,22,23,24,25,28,2A,2B,2C,2D and calculated episode duration less than 2 days);
- non-elective long stay (ADMIMETH=21,22,23,24,25,28,2A,2B,2C,2D and calculated episode duration greater than 1 day).

Mandatory data fields ('NEOCARE' neonatal level of care code, and 'CRITICALCARE_DAYS', 'REHABILITATION_DAYS', 'SPCDAYS' length of stay adjustments for critical care, rehabilitation and specialist palliative care) were not included in our HES extract, therefore left blank. All diagnosis codes had the following characters removed ('-', '&', '~', '+'), and ICD-10 codes I48X, L89X, Z22.5, K85X and H54.7 were replaced with I48.9, L89.9, Z22.9, K85.9 and H54.9 respectively. This was to allow comparisons year on year following changes to the underlying ICD10 classification and prevent the now invalid codes for U-grouping.

We also applied excess bed day costs to elective inpatient and non-elective long-stay episodes, for each day above the respective HRG Reference Cost trim point. Costs associated with unbundled HRGs were not included within the calculated admission costs.

In taking this approach, approximately 1% of the HES extract did not have an assigned HRG code resulted (considered ungrouped, and code UZ01Z assigned). Quality issues impacting failure to group into valid HRG codes also included poorly coded primary diagnoses (e.g. R69 "Unknown and unspecified causes of morbidity"), invalid secondary diagnoses (e.g. I84 "Haemorrhoids" which was available in earlier editions of ICD10 but not available in current 2016 version) and administrative errors (e.g. episode number not being unique in the spell/admission).

## Statistics

For the economic analyses, each data frame had two rows per patient (one for the pre phase and one for the post phase). Each row had a response variable (the number of observed admissions, the number of days in hospital, or the hospitalization cost), an offset (the duration of follow-up for that phase), a fixed effect (the phase: pre- or post) and a random effect (patient ID).

We estimated incident rate ratios in R using the 'glmer' function from the 'lme4' package with a Poisson family and log link function. Each patient had a random intercept. We estimated ratios of days hospitalized and costs using the 'cpglmm' function from the 'cplm' package, with a random intercept for each patient, and using the 'glmer' function with a Gamma distribution family and excluding patients with zero overnight admissions or zero costs. The mixed effects approach accounts for the lack of independence between samples.

For all models, the effect size (incidence rate ratio, ratio of days hospitalized, cost ratio) was the exponentiated value of the coefficient of the fixed variable, phase, and its confidence interval estimated from the standard error of the coefficient.

To account for some patients incurring zero in-patient cost, the distributions of hospitalized days and in-patient cost were fitted with Tweedie distributions [1]. These are a family of distributions with exponential dispersion, which are suitable for use in generalized linear models and account for the presence of a positive probability mass at zero. This approach is similar to the conventional practice of fitting costs with a Gamma distribution, but accounts for the presence of some patients incurring zero cost or in-patient days. Parameters of Tweedie distributions were fitted with maximum likelihood estimates using the R package 'tweedie^’^ [2].

We summarized observed hospitalized days and in-patient costs as means and SDs for each period. We estimated ratios and their 95% confidence intervals using compound Poisson generalized linear mixed models with R package 'cplm' [3], with a random intercept for each patient. These mixed-effects models fitted Tweedie distributions to the observed costs and days hospitalized. We estimated odds ratios of having at least one overnight stay and of incurring any cost with generalized binomial multilevel models with random intercepts for each patient. We used generalized multilevel gamma regression models with random intercepts for each patient to estimate ratios of days hospitalized for admitted patients and costs for those who incurred costs.

## References

1. Tweedie MCK. An index which distinguishes between some important exponential families. In: Ghosh JK, Roy J, editors. Statistics: Applications and new directions. Calcutta: Indian Statistical Institute; 1984. pp. 579–604.

2. Dunn PK, Smyth GK. Evaluation of Tweedie exponential dispersion model densities by Fourier inversion. Stat Comput. 2008;18: 73–86. doi:10.1007/s1122200790396

3. Zhang Y. Likelihood-based and Bayesian methods for Tweedie compound Poisson linear mixed models. Stat Comput. 2013;23: 743–757.
