# Supplementary material for: Safety, effectiveness and costs of percutaneous mitral valve repair: A real-world prospective study
Source: PLoS One. 2021 May 12;16(5):e0251463. doi: 10.1371/journal.pone.0251463 (PMC8115844; doi:10.1371/journal.pone.0251463)
Supplement: S1 Table — (DOCX) [file pone.0251463.s002.docx]

## S1 Table. List of outcome measures used in the registry.

| **Type** | **Outcome measure** | **Times** |
| --- | --- | --- |
| Efficacy | 1 or more clips successfully deployed | At procedure |
|  | Change in MR grade^a^ (from baseline) | FU (6w, 6m, 1y 2y) |
|  | Change in NYHA score (from baseline) | FU (6w, 6m, 1y 2y) |
|  | Change in EQ-5D (from baseline) | FU (6w, 6m, 1y 2y) |
|  | Change in Quality of Life VAS (from baseline) | FU (6w, 6m, 1y 2y) |
|  | Change in KCCQ score (from baseline) | FU (6w, 6m, 1y 2y) |
|  | Change in CCS angina status (from baseline) | FU (6w, 6m, 1y 2y) |
|  | Change in BNP (from baseline) | FU (6w, 6m, 1y 2y) |
|  | Change in six minute walk test (from baseline) | FU (6w, 6m, 1y 2y) |
|  | Subsequent mitral valve intervention | FU |
| Safety | Mortality | At procedure, FU |
|  | Major complications^b^ | At procedure, FU |
|  | Minor complications^c^ | At procedure, FU |
| Economic | Length of stay | At procedure |
|  | Change in utility (from baseline) | FU (1 year) |
|  | Admissions | Before procedure (1 year), after discharge (1 year) |
|  | Total all-cause hospitalisation days | Before procedure (1 year), after discharge (1 year) |
|  | Healthcare resource usage | Before procedure (1 year), after discharge (1 year) |
| Composite | Procedural success rate^d^ | At discharge |
| Abbreviations: FU, follow-up; LoS, length of stay; MR, mitral regurgitation; NYHA, New York Heart Association; HRQoL, health-related quality of life; VAS, visual analogue scale; KCCQ, Kansas City Cardiomyopathy Questionnaire.  ^a^MR Grade: Grade 1 (Mild): small central jet <4cm2 or <20% of LA area, vena contracta width <0.3cm, no or minimal flow convergence, regurgitant volume </= 30ml, EROA<20mm2; Grade 2 (Mild-Moderate): signs of MR >mild present, but no criteria for severe MR, EROA 20-29mm2, Regurgitant volume 30-44ml; Grade 3 (Moderate - severe) as Grade 2 but EROA 30-39mm2, Regurgitant volume 45-59ml; Grade 4 (Severe): vena contracta width >/= 0.7cm with large central MR jet (area >40% of LA), or with a wall-impinging jet of any size, swirling in LA; large flow convergence, systolic reversal in pulmonary veins, EROA ≥40mm2, regurgitant volume ≥60ml  ^b^Death; Neurological event; Additional surgery; Device embolisation (percutaneous retrieval); Myocardial infarction; Endocarditis; Pericardial effusion/tamponade (requiring intervention); Major vascular injury (requiring intervention); mitral valve complication; Oesophageal rupture; Major bleed; Acute Kidney Injury (stage 2/3); Cardiogenic shock.  ^c^Minor complications: Device failure; Partial detachment; Pericardial effusion/tamponade (treated conservatively); Thrombus; New moderate/severe mitral stenosis; Minor bleed; Acute Kidney Injury (stage 1); Minor vascular complication.  ^d^Device successfully implanted with no major complications. | | |
