# Supplementary material for: Safety, effectiveness and costs of percutaneous mitral valve repair: A real-world prospective study
Source: PLoS One. 2021 May 12;16(5):e0251463. doi: 10.1371/journal.pone.0251463 (PMC8115844; doi:10.1371/journal.pone.0251463)
Supplement: S2 Table — (DOCX) [file pone.0251463.s003.docx]

## S2 Table. Adverse events (in-hospital and following discharge).

|  | **In-hospital**  **(n=199)** | | **After discharge**  **(6w, 6m, 1y, 2y combined)**  **(n=170)** | |
| --- | --- | --- | --- | --- |
|  | **Total no. of procedures (multiple events permitted)** | **% [95% CI]** | **Total no. of procedures (multiple events permitted)** | **% [95% CI]** |
| **Major complications:** | **16** | **8.2 [4.7:12.9]** | **25** | **14.7 [9.7:20.9]** |
| Death | 10 | 5.1 [2.5:9.2] | 20 | 11.8 [7.3:17.6] |
| Neurological event | 1 | 0.5 [0.0:2.8] | 2 | 1.2 [0.1:4.2] |
| Additional surgery | 3 | 1.5 [0.3:4.5] | 4 | 2.4 [0.6:5.9] |
| Device embolisation (percutaneous retrieval) | 1 | 0.5 [0.0:2.9] | 0 | 0.0 [0.0:2.1] |
| MI | 2 | 1.0 [0.1:3.7] | 0 | 0.0 [0.0:2.1] |
| Endocarditis | 0 | 0.0 [0.0:1.9] | 0 | 0.0 [0.0:2.1] |
| Pericardial effusion/tamponade (requiring intervention) | 0 | 0.0 [0.0:1.9] | N/A | N/A |
| Major vascular injury (requiring intervention) | 0 | 0.0 [0.0:1.9] | N/A | N/A |
| MV complication | 0 | 0.0 [0.0:1.9] | N/A | N/A |
| Oesophageal rupture | 1 | 0.5 [0.0:3.0] | N/A | N/A |
| Major bleed | 3 | 1.6 [0.3:4.6] | N/A | N/A |
| AKI (stage 2/3) | 4 | 2.1 [0.6:5.3] | N/A | N/A |
| Cardiogenic shock | 2 | 1.1 [0.1:3.9] | N/A | N/A |
| **Minor complications:** | **15** | **7.6 [4.3:12.2]** | **22** | **12.9 [8.30:18.9]** |
| Device failure | 0 | 0.0 [0.0:1.9] | N/A | N/A |
| Partial detachment | 1 | 0.5 [0.0:2.9] | 1 | 0.6 [0.0:3.2] |
| Pericardial effusion/tamponade (treated conservatively) | 3 | 1.6 [0.3:4.5] | N/A | N/A |
| Thrombus | 0 | 0.0 [0.0:1.9] | N/A | N/A |
| New moderate/severe mitral stenosis | 3 | 1.8 [0.4:5.2] | 21 | 12.4 [7.8:18.3] |
| Minor bleed | 7 | 3.7 [1.5:7.5] | N/A | N/A |
| AKI (stage 1) | 1 | 0.5 [0.0:3.0] | N/A | N/A |
| Minor vascular complication | 0 | 0.0 [0.0:1.9] | N/A | N/A |
| **Any complication** | **30** | **15.2 [10.5:20.9]** | **44** | **25.9 [19.5:33.1]** |
| **Device implanted** | **187** | **94.0 [89.7:96.8]** | N/A | N/A |
| **Procedural success (device implanted in absence of major complications)** | **171** | **85.9 [80.3:90.4]** | N/A | N/A |
| **New requirement for permanent pacing** | **3** | **1.6 [0.3:4.7]** | N/A | N/A |
| N/A Not applicable | | | | |
