# Supplementary material for: Safety, effectiveness and costs of percutaneous mitral valve repair: A real-world prospective study
Source: PLoS One. 2021 May 12;16(5):e0251463. doi: 10.1371/journal.pone.0251463 (PMC8115844; doi:10.1371/journal.pone.0251463)
Supplement: S3 Table — (DOCX) [file pone.0251463.s004.docx]

## S3 Table. MR grade over 2 years follow up

|  | Time since procedure  Number (%) | | | | | | |
| --- | --- | --- | --- | --- | --- | --- | --- |
| MR grade | Pre-procedure | Post-procedure | Discharge | 6 weeks | 6 months | 1 year | 2 years |
| None (0) | 0 (0) | 21 (11.8) | 38 (21.8) | 2 (1.4) | 2 (1.9) | 1 (1.5) | 0 (0) |
| Mild (1+) | 0 (0) | 102 (57.3) | 60 (34.5) | 47 (32.6) | 35 (32.7) | 25 (36.8) | 9 (60.0) |
| Mild-moderate (2+) | 1 (0.5) | 43 (24.2) | 59 (33.9) | 61 (42.4) | 50 (46.7) | 25 (36.8) | 3 (20.0) |
| Moderate-severe (3+) | 19 (10.2) | 10 (5.6) | 13 (7.5) | 25 (17.4) | 16 (15.0) | 13 (19.1) | 3 (20.0) |
| Severe (4+) | 167 (89.3) | 2 (1.1) | 4 (2.3) | 9 (6.2) | 4 (3.7) | 4 (5.9) | 0 (0) |
| **p-value**  **(n pairs)** | **Reference** | **p<0.0001 (n=178)** | **p=0.0005**  **(n=174)** | **p<0.0001**  **(n=144)** | **p<0.0001**  **(n=107)** | **p<0.0001**  **(n=68)** | **p<0.0001**  **(n=15)** |
