# Supplementary material for: Safety, effectiveness and costs of percutaneous mitral valve repair: A real-world prospective study
Source: PLoS One. 2021 May 12;16(5):e0251463. doi: 10.1371/journal.pone.0251463 (PMC8115844; doi:10.1371/journal.pone.0251463)
Supplement: S4 Table — (DOCX) [file pone.0251463.s005.docx]

## S4 Table. NYHA class over 2 years follow up.

|  | Time since procedure  Number (%) | | | | |
| --- | --- | --- | --- | --- | --- |
| NYHA class | Pre-procedure | 6 weeks | 6 months | 1 year | 2 years |
| No limitation (I) | 3 (1.6) | 28 (18.8) | 27 (23.7) | 17 (23.3) | 7 (46.7) |
| Slight limitation (II) | 11 (5.9) | 94 (63.1) | 67 (58.8) | 41 (56.2) | 8 (53.3) |
| Marked limitation (III) | 118 (63.4) | 25 (16.8) | 20 (17.5) | 14 (19.2) | 0 (0.0) |
| Symptoms at rest (IV) | 54 (29.0) | 2 (1.3) | 0 (0.0) | 1 (1.4) | 0 (0.0) |
| Total patients | **186** | **149** | **114** | **73** | **15** |
