# Supplementary material for: Safety, effectiveness and costs of percutaneous mitral valve repair: A real-world prospective study
Source: PLoS One. 2021 May 12;16(5):e0251463. doi: 10.1371/journal.pone.0251463 (PMC8115844; doi:10.1371/journal.pone.0251463)
Supplement: S5 Table — S5a. HRQoL data (EQ-5D) over 2 years follow up. S5b. EQ-5D VAS over 2 years follow up. (DOCX) [file pone.0251463.s006.docx]

## S5a Table. HRQoL data (EQ-5D) over 2 years follow up.

| **Time point** | **Median utility score (Q1, Q3 quartiles)**  **Number of participants, n** | **Mean utility score (SD)** | **Mean change in utility score (SD)** | **Statistical significance compared with baseline (paired test)** |
| --- | --- | --- | --- | --- |
| Baseline (reference) | 0.6 (0.45, 0.72)  163 | 0.55 (0.23) | Reference | Reference |
| 6 weeks | 0.78 (0.66, 0.88)  136 | 0.75 (0.21) | 0.18 (0.23)  n=136 | p < 0.0001 |
| 6 months | 0.81 (0.71, 0.88)  117 | 0.77 (0.20) | 0.20 (0.24)  n=113 | p < 0.0001 |
| 1 year | 0.76 (0.64, 0.88)  56 | 0.73 (0.23) | 0.14 (0.22)  n=54 | p < 0.0001 |
| 2 years | 0.74 (0.69, 0.84)  12 | 0.76 (0.14) | 0.16 (0.23)  n=12 | p = 0.0393 |

## S5b Table. EQ-5D VAS over 2 years follow up.

| **Time point** | **Median VAS score, mm (Q1, Q3 quartiles)**  **Number of participants, n** | **Mean change in VAS score, mm (SD)**  **Number of participants, n** | **Statistical significance compared with baseline (paired test)** |
| --- | --- | --- | --- |
| Baseline (reference) | 50 (35, 65)  141 | Reference | Reference |
| 6 weeks | 70 (50, 80)  105 | 16.8 (18.9)  n=102 | p < 0.0001 |
| 6 months | 70 (60, 80)  88 | 21.4 (25.4)  n=85 | p < 0.0001 |
| 1 year | 75 (55, 85)  52 | 18.6 (24.2)  n=49 | p < 0.0001 |
| 2 years | 70 (60, 76) [20-90]  13 | 8.6 (36.0)  n=12 | p = 0.4265 |
