# Supplementary material for: Safety, effectiveness and costs of percutaneous mitral valve repair: A real-world prospective study
Source: PLoS One. 2021 May 12;16(5):e0251463. doi: 10.1371/journal.pone.0251463 (PMC8115844; doi:10.1371/journal.pone.0251463)
Supplement: S6 Table — (DOCX) [file pone.0251463.s007.docx]

## S6 Table. HRQoL disease-specific data (KKCQ) over 2 years follow up.

| **Time point** | **Median utility score (Q1, Q3 quartiles)**  **Number of participants n** | **Mean change in utility score (SD)**  **Number of participants n** | **Statistical significance compared with baseline (paired test)** |
| --- | --- | --- | --- |
| Baseline (reference) | 33.3 (16.7, 50.0)  159 | Reference | Reference |
| 6 weeks | 75.0 (50.0, 91.7)  137 | 34.9 (25.6)  n=134 | NR? |
| 6 months | 83.3 (58.3, 91.7)  115 | 37.5 (27.9)  n=109 | NR? |
| 1 year | 75.0 (50.0, 91.7)  61 | 33.2 (28.8)  n=57 | NR? |
| 2 years | 41.7 (33.3, 50.0  13 | 3.8 (24.8)  n=11 | NR? |
