# Supplementary material for: Safety, effectiveness and costs of percutaneous mitral valve repair: A real-world prospective study
Source: PLoS One. 2021 May 12;16(5):e0251463. doi: 10.1371/journal.pone.0251463 (PMC8115844; doi:10.1371/journal.pone.0251463)
Supplement: S7 Table — (DOCX) [file pone.0251463.s008.docx]

## S7 Table. Hospital resource use before and after PMVR, stratified by reason for readmission.

| Outcome | Pre-procedural phase | Post-procedural phase | Rate ratio (95% CI) | p value |
| --- | --- | --- | --- | --- |
| *All patients (n = 150)* | | | | |
| Admissions – all | 470 | 251 | 0.57 (0.49 to 0.67) | <0.001 |
| Admissions – NC | 119 | 151 | 1.40 (1.10 to 1.79) | 0.00655 |
| Admissions – C | 351 | 100 | 0.30 (0.24 to 0.038) | <0.001 |
| Admissions - HF | 195 | 45 | 0.24 (0.18 to 0.34) | <0.001 |
| Days in hospital - all | 1635 | 1340 | 0.73 (0.54 to 0.98) | N/A |
| Days in hospital – NC | 338 | 689 | 1.62 (0.96 to 2.75) | N/A |
| Days in hospital – C | 1297 | 651 | 0.45 (0.31 to 0.64) | N/A |
| Days in hospital - HF | 784 | 449 | 0.53 (0.32 to 0.86) | N/A |
| Total costs (GBP) - all | 1,267,321 | 889,721 | 0.62 (0.50 to 0.79) | N/A |
| Total costs (GBP) - NC | 253,261 | 379,176 | 1.42 (0.96 to 2.12) | N/A |
| Total costs (GBP) – C | 1,014,060 | 510,545 | 0.41 (0.31 to 0.54) | N/A |
| Total costs (GBP) - HF | 597,322 | 320,061 | 0.39 (0.27 to 0.58) | N/A |
| *Surviving patients (n =133)** | | | | |
| Admissions – all | 418 | 221 | 0.53 (0.45 to 0.62) | <0.001 |
| Admissions – NC | 98 | 136 | 1.39 (1.07 to 1.80) | 0.0134 |
| Admissions – C | 320 | 85 | 0.27 (0.21 to 0.34) | <0.001 |
| Admissions - HF | 177 | 38 | 0.21 (0.15 to 0.30) | <0.001 |
| Days in hospital - all | 1432 | 1092 | 0.67 (0.49 to 0.93) | N/A |
| Days in hospital – NC | 259 | 549 | 1.71 (0.98 to 3.00) | N/A |
| Days in hospital – C | 1173 | 543 | 0.40 (0.27 to 0.60) | N/A |
| Days in hospital - HF | 677 | 369 | 0.50 (0.29 to 0.87) | N/A |
| Total costs (GBP) - all | 1,130,425 | 743,260 | 0.57 (0.45 to 0.73) | N/A |
| Total costs (GBP) - NC | 189,163 | 311,188 | 1.54 (1.01 to 2.35) | N/A |
| Total costs (GBP) – C | 941,261 | 432,072 | 0.36 (0.26 to 0.48) | N/A |
| Total costs (GBP) - HF | 543,814 | 273,808 | 0.35 (0.23 to 0.52) | N/A |
| Abbreviations: C, cardiac; GBP, British pound sterling; HF, heart failure; N/A, not applicable, NC, non-cardiac.  *Sensitivity analysis was performed where patients who had died post-procedure were excluded from analysis. This was done to address the “positive” economic consequences of healthcare resource reduction due to death, bearing in mind the high mortality rate associated with the procedure | | | | |
