# Supplementary material for: Safety, effectiveness and costs of percutaneous mitral valve repair: A real-world prospective study
Source: PLoS One. 2021 May 12;16(5):e0251463. doi: 10.1371/journal.pone.0251463 (PMC8115844; doi:10.1371/journal.pone.0251463)
Supplement: S8 Table — S8a. Comparison of demographics, inclusion and exclusion criteria with the MITRA-FR study [18]. Shaded rows indicate potentially important differences. S8b. Comparison of demographics, inclusion and exclusion criteria with the intervention arm of the COAPT study [19]. Shaded rows indicate potentially important differences. S8c. Comparison of demographics, inclusion and exclusion criteria with the EVEREST II/REALISM Continued Access Registry [13]. Shaded rows indicate potentially important differences. (DOCX) [file pone.0251463.s009.docx]

Table S8a. Comparison of demographics, inclusion and exclusion criteria with the MITRA-FR study [18]. Shaded rows indicate potentially important differences.

|  | MITRA-FR | This study |
| --- | --- | --- |
| *Inclusion criteria* | | |
| MR aetiology | 100% secondary MR (FMR) | Aetiology was not an inclusion criterion; secondary MR (FMR) 60%, primary (DMR) 40% |
| MR grade/severity | Severe secondary mitral regurgitation with regurgitation volume greater than 30ml per beat or an effective regurgitant orifice area (EROA) of greater than 20mm^2^ as assessed by echocardiography in accordance with 2012 guideline on the European Society of Cardiology | MR grade 2,3,4; Grade 2 included regurgitation volume 30-44ml, EROA of 20-29mm^2^; Grade 3 regurgitant volume 45-59ml, EROA 30-39mm^2^; Grade 4 regurgitant volume ≥60ml, EROA ≥40mm^2^. |
| LVEF | 15-40% | LVEF was not an inclusion criterion. 58.1% had an LVEF of 45% or greater; 18.6% LVEF 45-54%, 39.5% LVEF ≥55%. |
| NYHA Class | II, III, IV | NYHA was not an inclusion criterion; only 3 patients (1.5%) had NYHA Class I |
| *Exclusion criteria* | | |
| Eligible for surgery | Candidates for mitral-valve surgery | Included high-risk for surgery with or without offering bail-out |
| Laboratory criteria | Patients were excluded if they did not meet core laboratory criteria | Lab results were not an inclusion criterion |
| *Demographics* | | |
| Age, mean (SD), years | 70.1 (10.1) | 76.2 (10.5) |
| Male, n (%) | 120 (78.9%) | 137 (68.8%) |
| Diabetes | 50 (32.9%) | 36 (18.1%) |
| Previous MI | 75 (49.3%) | 79 (40.3%) |
| Heart rate, mean (SD) bpm | 73 (13) | 74 (15.7)* |
| Logistic EuroScore II, median (IQR) | 6.6 (3.5-11.9) | 4.8 (3.0-7.6) |
| Abbreviations:  MR: mitral regurgitation  FMR: functional (secondary) MR  DMR: degenerative (primary) MR  LVEF: Left ventricular ejection fraction  NYHA: New York Heart Association functional classification of heart failure  MI: myocardial infarction  SD: standard deviation  IQR: inter-quartile range  *at time of echo | | |

Table S8b. Comparison of demographics, inclusion and exclusion criteria with the intervention arm of the COAPT study [19]. Shaded rows indicate potentially important differences.

|  | COAPT | This study |
| --- | --- | --- |
| Inclusion criteria | | |
| MR aetiology | 100% secondary MR (FMR) | Aetiology was not an inclusion criterion; secondary MR (FMR) 60%, primary (DMR) 40% |
| LVEF | 20-50% | LVEF was not in an inclusion criterion. 58.1% had an LVEF of 45% or greater; 18.6% LVEF 45-54%, 39.5% LVEF ≥55%. |
| MR grade | Grade III (49%) or IV (51%) (confirmed by echo) | Grade II 0.5%, III 10.1%, IV 89.4% |
| NYHA class | Symptomatic despite maximal doses of medical therapy and cardiac resynchronisation therapy if appropriate (NYHA class II-IVa); NYHA class I 0.3%, II 42.7%, III 51.0%, IVa 6.0% | NYHA not an inclusion criterion; 3 patients (1.5%) had class I; II 6.1%, III 62.6%, IV 29.8% |
| Exclusion criteria | | |
| Eligible for surgery | Mitral valve surgery appropriate | Included high-risk for surgery with or without offering bail-out |
| Demographics | | |
| Age, mean (SD) years | 71.7 (11.8) | 76.2 (10.5) |
| Male, n (%) | 201 (66.6%) | 137 (68.8%) |
| Diabetes | 106 (35.1%) | 36 (18.1%) |
| Hypertension | 243 (80.5%) | 104 (53.3%) |
| Previous MI | 156 (51.7%) | 79 (40.3%) |
| Previous PCI | 130 (43.0%) | 45 (23.1%) |
| Abbreviations:  MR: mitral regurgitation  FMR: functional (secondary) MR  DMR: degenerative (primary) MR  LVEF: Left ventricular ejection fraction  NYHA: New York Heart Association functional classification of heart failure  MI: myocardial infarction  PCI: percutaneous coronary intervention  SD: standard deviation  IQR: inter-quartile range | | |

Table S8c. Comparison of demographics, inclusion and exclusion criteria with the EVEREST II/REALISM Continued Access Registry [13]. Shaded rows indicate potentially important differences.

|  | EVEREST II/ REALISM | This study |
| --- | --- | --- |
| *Inclusion criteria* | | |
| MR grade | Grades 3+ to 4+; Population was grade 2+ (13.6%), grade 3+ (52.4%), grade 4+ (25.8%), 8.2% unknown. | Patients were required to have symptomatic severe grade 3+ or 4+ MR. population was grade 2 0.5%, grade 3 10.1%, grade 4 89.4%. |
| Cardiac anatomy suitable for MitraClip | The primary regurgitant jet had to originate from leaflet  malcoaptation at the A2/P2 region. | Have suitable cardiac anatomy. |
| *Exclusion criteria* | | |
| Recent MI | MI within 2 weeks | Not defined. |
| LVEF | < 20%; Population mean (SD) 49.6% (14.0%) | LVEF was not an inclusion criterion. Population: 58.1% had an LVEF of 45% or greater; 18.6% LVEF 45-54%, 39.5% LVEF ≥55%. |
| Left ventricular end-systolic dimension (LVIDs) | >60mm | LVIDs was not an inclusion criterion. Population median (IQR): 43 (32:50), range 18 to 76 mm |
| MV area | <4.0cm^2^ | MV area not recorded |
| Eligible for surgery | High risk for surgery, using STS criteria. | Included high-risk for surgery with or without offering bail-out |
| *Demographics* | | |
| MR aetiology | FMR 63.3%; DMR 36.7% | Secondary MR (FMR) 60%, primary (DMR) 40% |
| Age, mean (SD), years | 80 (7.2) | 76.2 (10.5) |
| Male, n (%) | 242 (60.0%) | 137 (68.8%) |
| Diabetes | 141 (35.2%) | 36 (18.1%) |
| Previous MI | 187 (47.5%) | 79 (40.3%) |
| NYHA Class | Class I/II (16.6%), class III (62.0%); class IV (21.3%) | 3 patients (1.5%) had class I; II 6.1%, III 62.6%, IV 29.8% |
| Abbreviations:  MI: myocardial infarction  MR: mitral regurgitation  FMR: functional (secondary) MR  DMR: degenerative (primary) MR  LVEF: Left ventricular ejection fraction  NYHA: New York Heart Association functional classification of heart failure  STS: Society of Cardiac Surgeons  SD: standard deviation  IQR: inter-quartile range | | |
