# Supplementary material for: Safety, effectiveness and costs of percutaneous mitral valve repair: A real-world prospective study
Source: PLoS One. 2021 May 12;16(5):e0251463. doi: 10.1371/journal.pone.0251463 (PMC8115844; doi:10.1371/journal.pone.0251463)

## S1 Fig. Kaplan-Meier analysis over 2 years follow up of (a) patients receiving elective treatment (b) patients receiving urgent or emergency treatment.


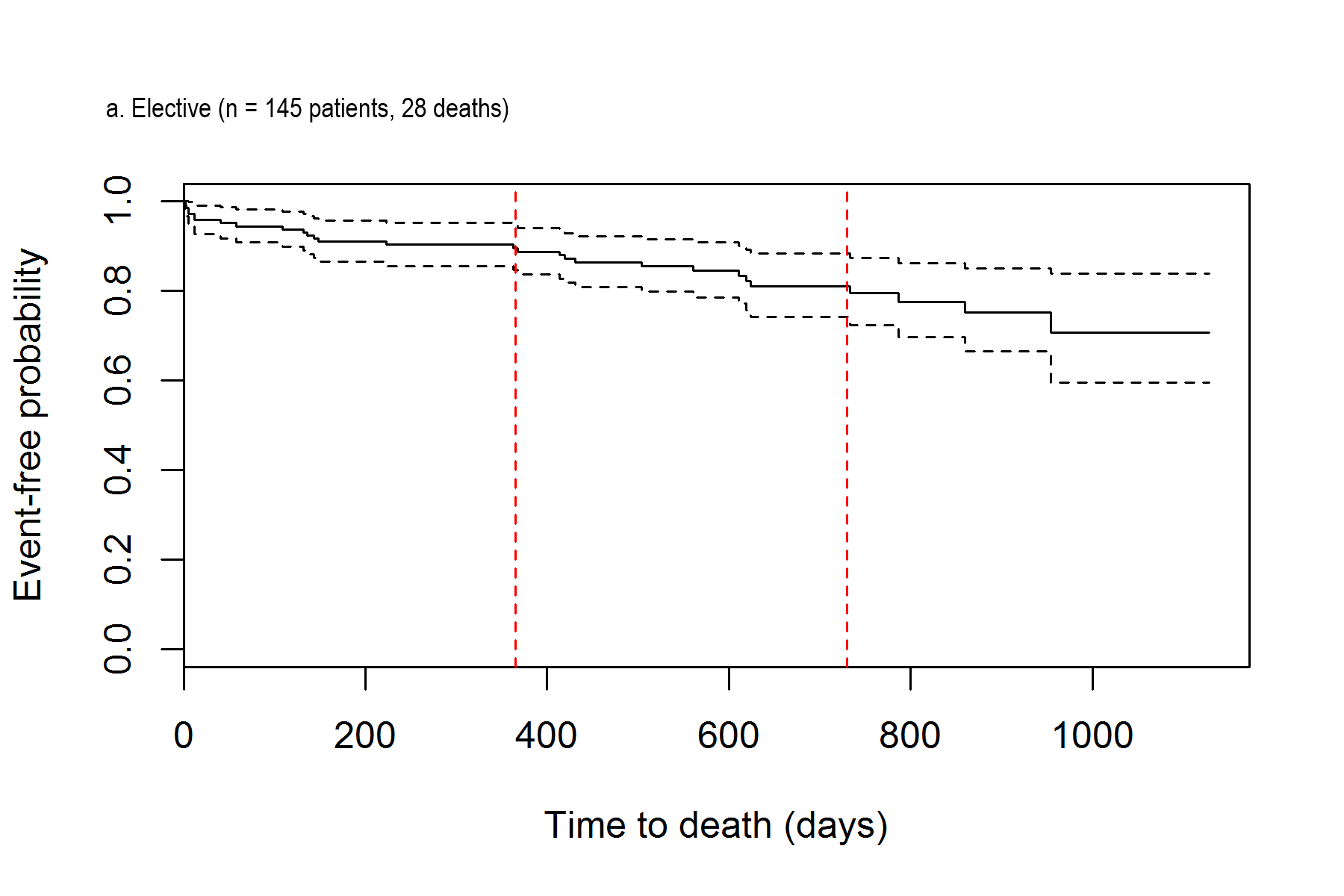


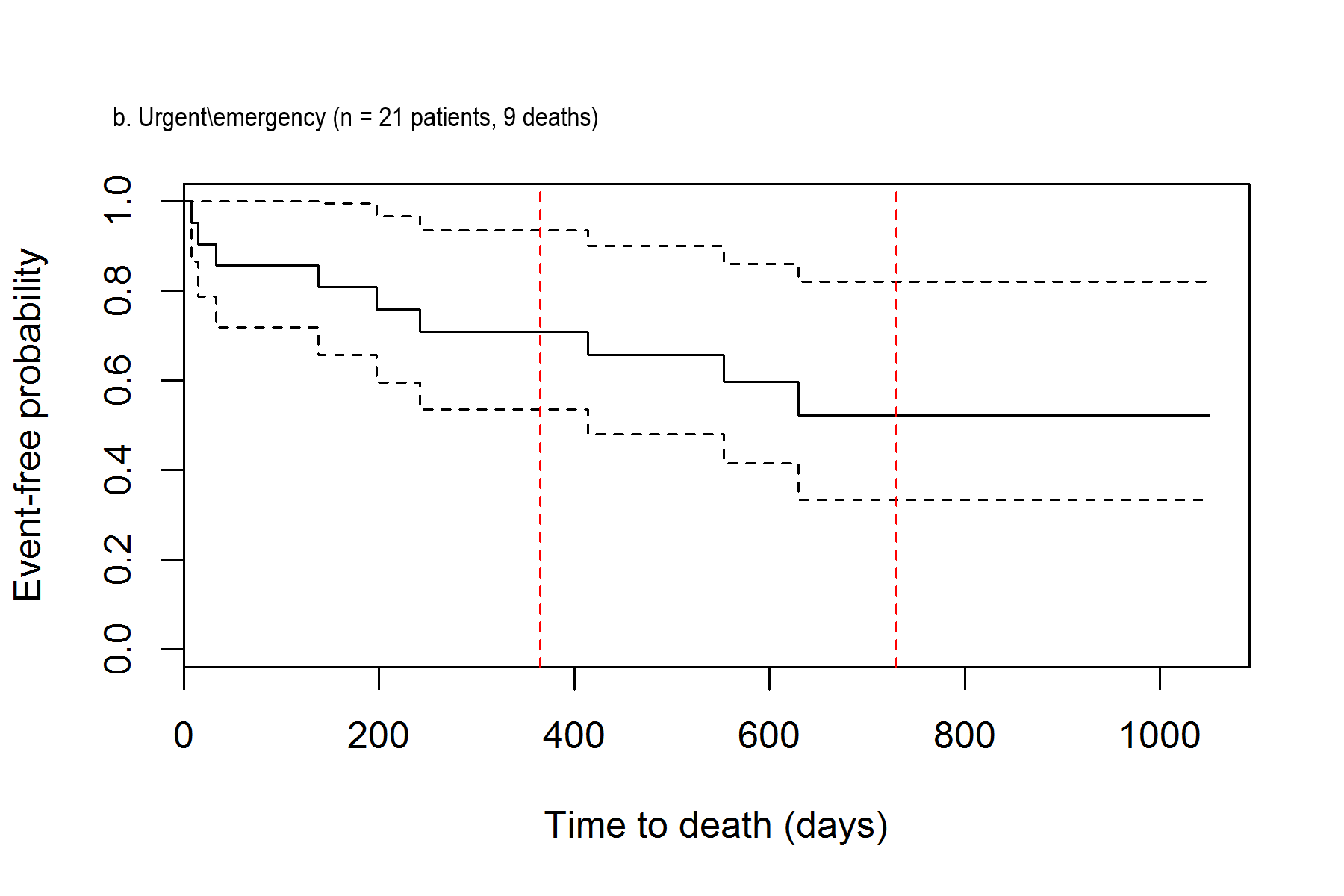

Supplement: S1 Fig — (DOCX) [file pone.0251463.s010.docx]
