# Supplementary material for: Safety, effectiveness and costs of percutaneous mitral valve repair: A real-world prospective study
Source: PLoS One. 2021 May 12;16(5):e0251463. doi: 10.1371/journal.pone.0251463 (PMC8115844; doi:10.1371/journal.pone.0251463)

## S2 Fig. Kaplan-Meier analysis over 2 years follow up of (a) patients with functional MR (b) patients with degenerative MR.


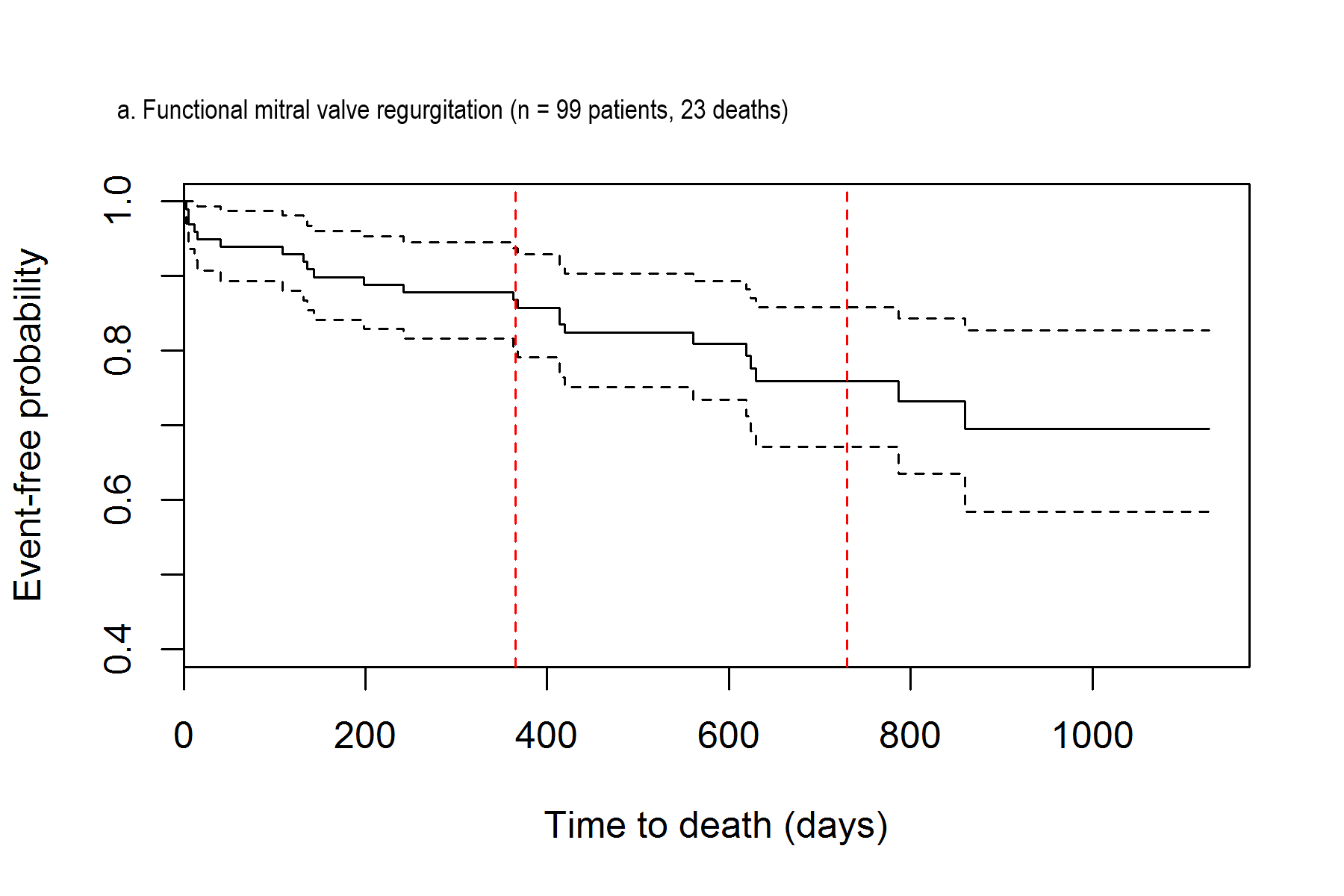


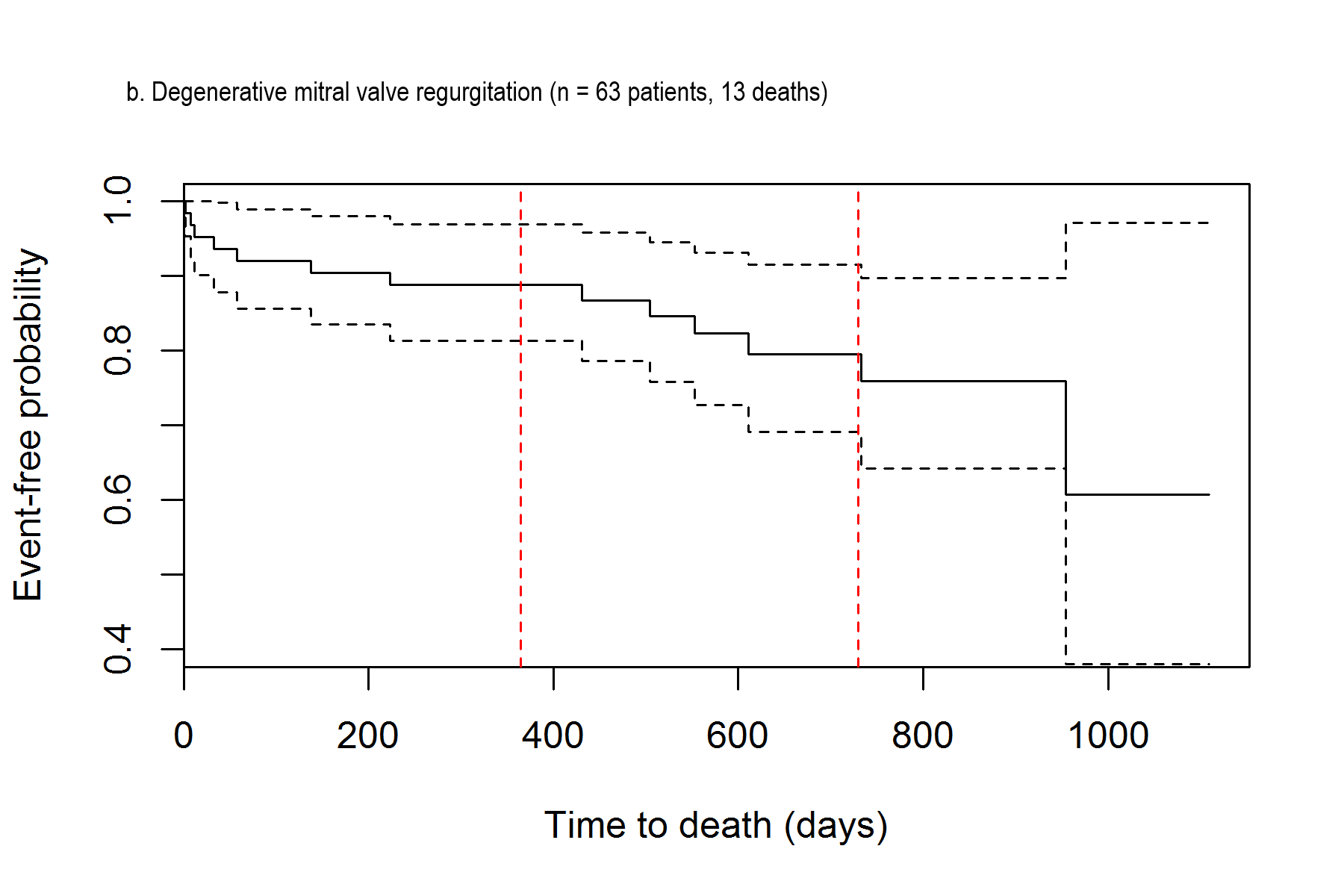

Supplement: S2 Fig — (DOCX) [file pone.0251463.s011.docx]
